# Mapping Lightscapes: Spatial Patterning of Artificial Lighting in an Urban Landscape

**DOI:** 10.1371/journal.pone.0061460

**Published:** 2013-05-06

**Authors:** James D. Hale, Gemma Davies, Alison J. Fairbrass, Thomas J. Matthews, Christopher D. F. Rogers, Jon P. Sadler

**Affiliations:** 1 School of Geography, Earth and Environmental Sciences, The University of Birmingham, Birmingham, West Midlands, United Kingdom; 2 Lancaster Environment Centre, Lancaster University, Lancaster, Lancashire, United Kingdom; 3 Centre for Urban Sustainability and Resilience, University College London, London, United Kingdom; 4 School of Geography and the Environment, University of Oxford, Oxford, Oxfordshire, United Kingdom; 5 School of Civil Engineering, The University of Birmingham, Birmingham, West Midlands, United Kingdom; University of Warwick, United Kingdom

## Abstract

Artificial lighting is strongly associated with urbanisation and is increasing in its extent, brightness and spectral range. Changes in urban lighting have both positive and negative effects on city performance, yet little is known about how its character and magnitude vary across the urban landscape. A major barrier to related research, planning and governance has been the lack of lighting data at the city extent, particularly at a fine spatial resolution. Our aims were therefore to capture such data using aerial night photography and to undertake a case study of urban lighting. We present the finest scale multi-spectral lighting dataset available for an entire city and explore how lighting metrics vary with built density and land-use. We found positive relationships between artificial lighting indicators and built density at coarse spatial scales, whilst at a local level lighting varied with land-use. Manufacturing and housing are the primary land-use zones responsible for the city’s brightly lit areas, yet manufacturing sites are relatively rare within the city. Our data suggests that efforts to address light pollution should broaden their focus from residential street lighting to include security lighting within manufacturing areas.

## Introduction

As the global population grows and becomes increasingly urban [1], [2], cities are increasing in their spatial extent [3], intensity of use [4] and physical heterogeneity [5]. Measuring variation within urban systems (in terms of their composition, configuration and function) plays a vital role in supporting research and management for improved sustainability performance [6], [7], [8], [9], [10]. However, systematic urban data collection and interpretation is challenging [11] given the high spatial variability within [12] and between urban areas [13], the co-variability between features of urbanisation [14] and scale dependent relationships [15]. Multiple and diverse measures of urbanisation at a range of spatial scales are therefore desirable [16].

One variable that is closely associated with urbanisation is outdoor artificial lighting. Remotely sensed data are good predictors of both urban extent [17], [18] and population size [19], [20] at coarse spatial scales. Like urbanisation, the spatial coverage and intensity of artificial light pollution appear to be increasing [21], [22]; whilst the spectrum of the night sky is also changing due to the replacement of lighting infrastructure [23]. Lighting has strong cultural links to ideas of modernity and safety [24] and is a hallmark of development, giving people greater choices as to where, when and how long their activities can take place. However, lighting has other direct effects on health [25], [26], culture and amenity [27], [28], [29], safety [30], security [31] and ecology [32], [33] and indirect effects on economics [34] and carbon emissions [35]. Given the broad sustainability implications of increases in artificial lighting, research programs are emerging that examine this phenomenon from a range of disciplinary perspectives (e.g. www.verlustdernacht.de). However, strategies and policies for the management of artificial lighting are less comprehensive than might be expected [24]. The lack of high resolution mapping of artificial lighting is increasingly recognised as an important barrier to related research and management [36]. Datasets exist globally at a coarse spatial (∼3 km) and spectral resolution, allowing broad variations in urban lighting to be detected [27]; but sub-city patterning cannot be explored effectively [36], [37]. Numerous colour photographs are available from the International Space Station with a spatial resolution of up to 60 m [36]. Although these images are starting to be used to detect demographic patterns within urban areas [38], individual lamps still cannot be identified [36]. Finer spatial resolution data do exist, but typically have a limited spatial extent [36], [39] (but see [40]). This hinders the development of a strong evidence base to support urban lighting strategies, as cities can be highly heterogeneous even at fine spatial scales [5], [9]. For example, little is known about how lighting varies with urban land-use [40], [41], [42] or along built density gradients. Improved baseline urban lighting maps are also needed in order to apply the results of published lighting research e.g. [43], to implement existing planning guidance on urban lighting zones [42], [44], to enforce planning consents and legislation related to lighting nuisance [45], [46] and to monitor changes over time. Therefore, there is a need to secure lighting datasets at the city scale; and at a spatial and spectral resolution sufficient to advance lighting research and the planning and governance of urban lighting.

In this study our aims were: 1) to develop a method for securing fine resolution urban lighting datasets and 2) to undertake a city case study exploring how lighting varies with built density and land-use. Here, we present the finest resolution multi-spectral night-time photograph of an entire city, processed to derive estimates of surface illuminance and the location and nature of individual lamps. We found positive relationships between artificial lighting indicators and built density at coarse spatial scales, whilst at a local level lighting varied significantly with land-use.

## Methods

### Data Collection and Processing

**Figure 1:**
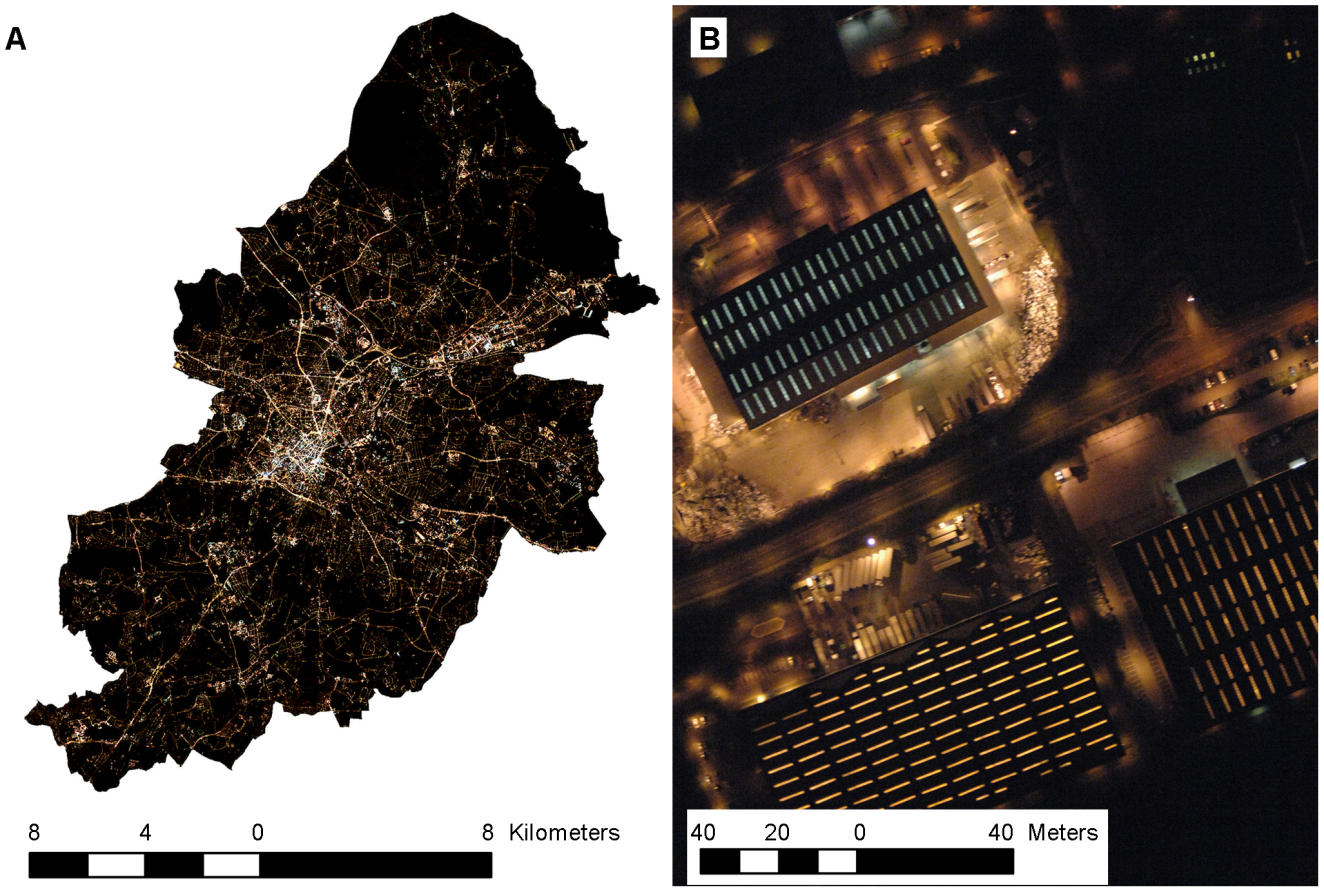
Aerial night photography examples. (A) The city of Birmingham and (B) a retail distribution centre. Reprinted from original aerial photography under a CC BY license, with permission from the Environment Agency, original copyright [2009].

Aerial night photography was collected in March 2009 by the UK Environment Agency, with support from the Birmingham Environmental Partnership (Fig. 1). The target area was Birmingham, a large city (268 km^2^) within the highly urbanised West Midlands metropolitan county of the United Kingdom. Surveys were undertaken by plane at a height of ∼900 m, using a colour Nikon D2X digital camera, a 24 mm AF Nikkor lens and a 1/100 ths exposure. The resulting RGB images were orthorectified, mosaiced and re-sampled from 10 cm to 1 m pixel resolution. This single image was then processed to derive two landscape indicators of artificial lighting: a raster layer representing incident surface lux and a point layer representing the location and class of individual lamps. These indicators were considered to be of broad interest for those studying and managing lighting in urban landscapes.

Field surveys of ground incident lighting were undertaken in order to develop these indicators, using a USB2000+VIS-NIR Spectrometer (Ocean Optics, Florida, USA). Surveys were stratified over a range of lamps types located in both dense urban and residential neighbourhoods. Starting below each lamp, ground measurements of incident lux (lx) were collected at 1 m intervals along a linear transect (total 400 measurements). Using a GIS (ArcGIS 9.2, ESRI Redlands, USA) these point survey data were superimposed onto a single band (greyscale) raster, generated by averaging pixel values from the RGB image of the city using ER Mapper 7.2 (ER Mapper, San Diego, USA). The pixel value below each point was then extracted, allowing the relationship between incident lux and pixel value to be modelled. Model fit was found to improve when the measurements taken between 0 and 2 m from the lamp were removed. This was likely due to inconsistent signal sources for the camera; in some cases the signal coming directly from unshielded lamps whilst in others from light reflected by the surfaces below a shielded lamp. The equation for the final model (Fig. S1) was then used to reclassify the greyscale raster to represent incident lux (hereafter referred to as the “lux layer”). To derive an estimate of noise we extracted raster summaries for 25 ha of the greyscale raster corresponding to areas of the landscape known to be unlit. For these “dark” locations, 99% of greyscale pixels had values of less than 20 (Fig. S2). Pixel values <20 were therefore considered to be unlit for the purposes of the landscape analysis. Three raster layers were generated representing areas lit to ≥10, ≥20 and ≥30lx.

To identify the point location of all lamps within the landscape, we used the focal statistics and raster calculator tools in ArcGIS to identify the brightest pixels at a processing resolution of 10 m. First, a focal maximum layer was created using a circular roving window of 10 m radius. The raster calculator tool was then used to identify pixels in this focal maximum layer whose values were identical to the original greyscale raster, which were then reclassified into a binary raster layer representing potential lamp locations (the candidate lamp layer). A 10 m sample radius was chosen because street lamps are typically spaced at greater intervals and it was also found that this reduced the occurrence of false lamp signals due to highly reflective surfaces. Although generating this layer succeeded in identifying lamp locations, a large proportion of the candidate lamp pixels did not correspond to a lamp. These were the result of small variations in greyscale pixel values within dark areas such as parks and gardens. To address this, statistics for a selection of confirmed lamp locations were compared to a sample of these “dark” locations. Focal statistics layers were created from the greyscale raster as well as from the individual red, green and blue layers of the mosaiced night photograph. These layers were generated using circular neighbourhoods of radii up to 7 m, as well as annuli that excluded the neighbourhood centre. Using a CHAID classification tree (SPSS 18.0), we found that the majority (95.4%) of locations representing lamp centres had average green pixel values between 1 m and 2 m from the lamp of ≥14 whilst the majority (99.8%) of locations within unlit areas had values for this measure of <14. This threshold was therefore used to remove dark locations within the candidate lamp layer and the remaining pixels were converted to a point layer representing 117,599 lamp centres within the city.

Elvidge et al [47], demonstrated the potential for discriminating major lamp types by using a 3 band sensor that broadly covered the visible light spectrum. Whilst the RGB bands in our image did not correspond exactly to the band widths proposed by Elvidge et al [47], we considered it feasible that they would be sufficient to differentiate between the major classes of street lamps present in the city: mercury vapour (MV), metal halide (MH), low pressure sodium (LPS) and high pressure sodium (HPS). Focal statistics were extracted for 240 lamp centres of known class and a CHAID classification tree was used to differentiate between lamp types (Fig. S3). The first discriminating variable was the green to red ratio (G:R) for pixels up to 1 m from the lamp centre. A G:R of 0.96 separated the orange lamps (LPS and HPS) from white lamps (MH and MV), with an accuracy of 98.3% in both cases. LPS and HPS lamps were then differentiated based on the maximum greyscale pixel value between 2 and 4 m from the lamp centre. Values < = 48 indicated an LPS lamp (96.7% correct), whilst HPS lamps typically had values >48 (81.7% correct) (Table 1). MH and MV were differentiated based on the average blue pixel value up to 1 m from the lamp centre. Values >33.2 gave a 93.3% correct classification for MH, whilst values < = 33.2 gave a 98.8% correct classification for MV. These thresholds were then used to classify all city lamp centres into the 4 broad lamp classes.

**Table 1:** Results of CHAID classification for lamp class.

Observed		Predicted lamp class				
Lamp class	Sample	LPS	HPS	MH	MV	Percent Correct
LPS	60	58	1	0	1	96.7%
HPS	60	10	49	0	1	81.7%
MH	60	1	0	56	3	93.3%
MV	60	1	0	0	59	98.3%

Classification of lamps using pixel values from the aerial night photograph, corresponding to individual lamp locations. Accuracy is estimated based on a sample of 60 known lamps for each lamp class. LPS = low pressure sodium, HPS = high pressure sodium, MH = metal halide, MV = mercury vapour.

### Landscape Analysis

**Figure 2:**
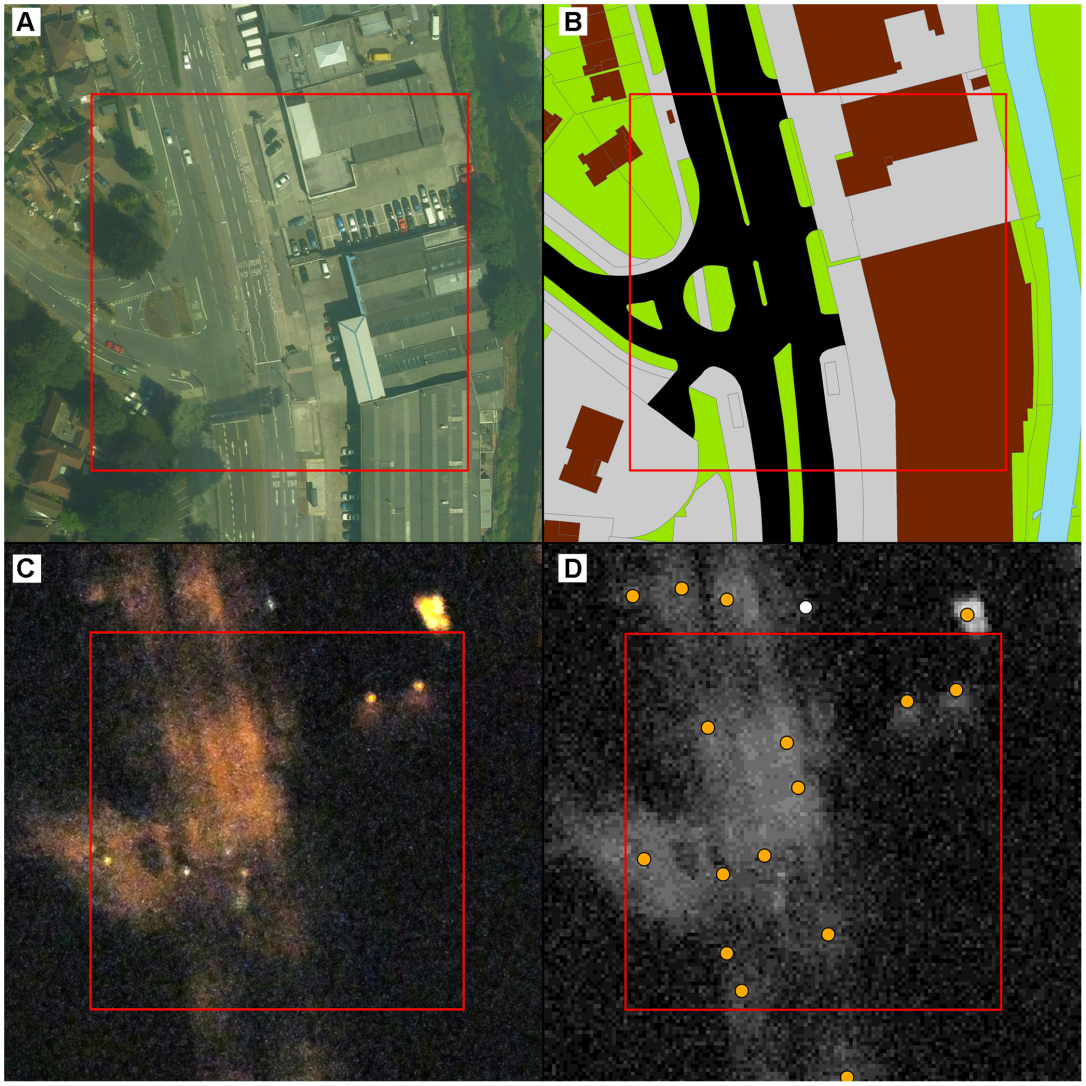
Aerial photography, mapping and lighting indicators for a 100 m square manufacturing zone and road intersection. (A) A daytime aerial photograph, reprinted from original photography under a CC BY license, with permission from Bluesky International Limited, original copyright [2007] (B) OS MasterMap land-cover and land-use parcels reprinted from original mapping under a CC BY license, with permission from the Ordinance Survey, original copyright [2008], (C) a night time aerial colour photograph reprinted from original aerial photography under a CC BY license, with permission from the Environment Agency, original copyright [2009] and (D) a raster representing ground lux, overlain by a point layer representing lamp centres.

The sampling strategy was intended to reflect key scales and boundaries of urban ownership, management and decision-making [12], [48]. GIS analyses were undertaken to explore patterns between two broad lighting metrics (lit area and number of lamps) and measures of urban composition. To explore the effect of urban density, Ordnance Survey MasterMap (OSMM) land-cover and land-use parcels that were dominated by built land-cover (e.g. roads, car parks and buildings but not gardens) were combined into a single “built” category. These were then converted to a 1 m resolution raster representing built land-cover for the entire city. Grid squares of increasing size (0.01 km^2^, 0.25 km^2^, 1 km^2^ and 4 km^2^) were then used to extract summaries of built land-cover and lighting metrics. Because broad urbanisation gradients typically fail to capture the effect of different land-uses [49], we employed a complementary approach to measuring urban performance by sampling the lighting of land-use units. Importantly, we used two contrasting land-use classifications to maximise the utility of the results (Table S1); National Land Use Database (NLUD) zones [50] and OSMM parcels. NLUD data included categories such as housing, manufacturing and education, which were available for the entire city as 100 m grid squares (0.01 km^2^). OSMM parcels were typically smaller than 0.01 km^2^ and irregularly shaped, representing features such as gardens, pavements and buildings. OSMM parcels were grouped to reflect five broad management categories (Table S1). Each 0.01 km^2^ NLUD land-use zone was therefore typically composed of a number of smaller OSMM land-use parcels (Fig. 2). Lighting indicator summaries were extracted for both the land-use zones and land-use parcels at the city scale. These were used to estimate the percentage contribution of different land-uses to the total number of lamps and total lit area within the city. In addition, we calculated the lamp density and percentage lit area for each land-use zone and parcel type. These provided an indication of how intensely lit different land-uses were, irrespective of how much they contributed to lighting at the city scale.

## Results

### Landscape Scale Patterns between Lighting and Built Density

**Figure 3:**
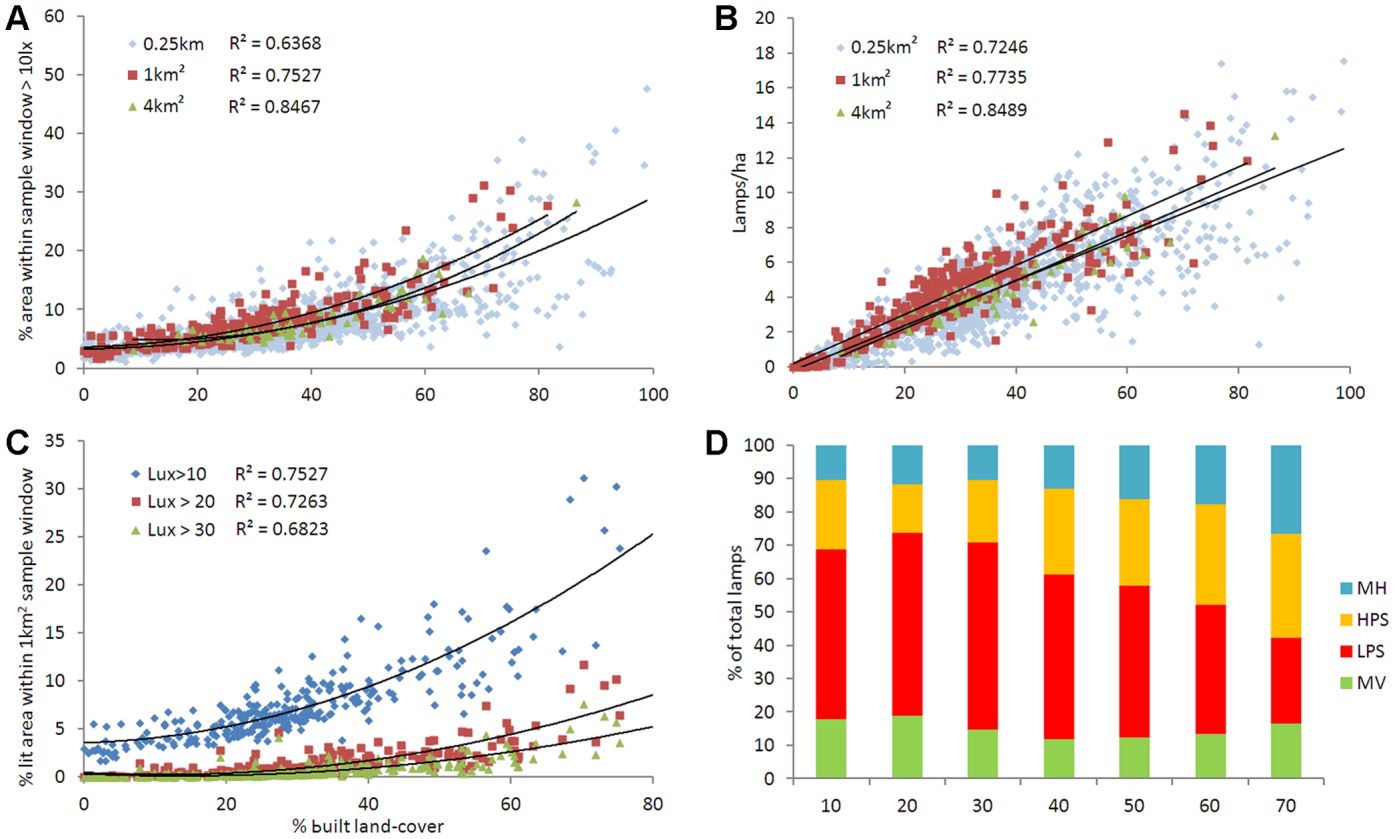
Percentage built land-cover plotted against a variety of lighting metrics. (A) Percentage lit area (≥10lx) sampled at 0.25 km^2^, 1 km^2^ and 4 km^2^ scales. (B) Density of lamps sampled at 0.25 km^2^, 1 km^2^ and 4 km^2^ scales. (C) Percentage lit area ≥10, ≥20 and ≥30lx at the 1 km^2^ scale. (D) Lamp class sampled at the 1 km^2^ scale. LPS = low pressure sodium, HPS = high pressure sodium, MH = metal halide, MV = mercury vapour. Y axis values are standardised as a percentage of the total number of lamps within each built density class. Built density values represent class maximum (10 = 0–10% built land cover).

8% of the total land surface of the city was found to be illuminated to at least 10lx. In addition, 65% of all lit surfaces (≥10lx) and 80% of all city lamps were directly associated with built land-cover. Lighting indicators demonstrate positive and often non-linear relationships with the density of built land-cover. The percentage of lit area increases in a non-linear fashion along these urbanisation gradients (Figs 3A & C), whilst lamp density increases linearly (Fig. 3B). As the scale of sampling (window size) increases, the fit of these models improves; although the relationships remain essentially the same (Fig. 3A & B). The results for sampling at the 0.01 km^2^ scale are presented for comparison in figure S4. The percentage of each sample square that is lit to ≥10lx rises from ∼5% in rural or semi-natural areas to ∼30% in densely built areas (Fig. 3A). Similarly, lamp density rises from ∼0 lamps/ha in rural or semi-natural areas to ∼15/ha in densely built areas (Fig. 3B). The composition of lamp types also changes along the 1 km^2^ urban gradient (Fig. 3D), with LPS lamps dominating provision at low built densities, shifting to (broader spectrum) HPS and MH lamps in densely built areas. Changes in the density of individual lamp types along the 1 km^2^ urban gradient are presented in figure S5.

### Lighting and Land-use

**Figure 4:**
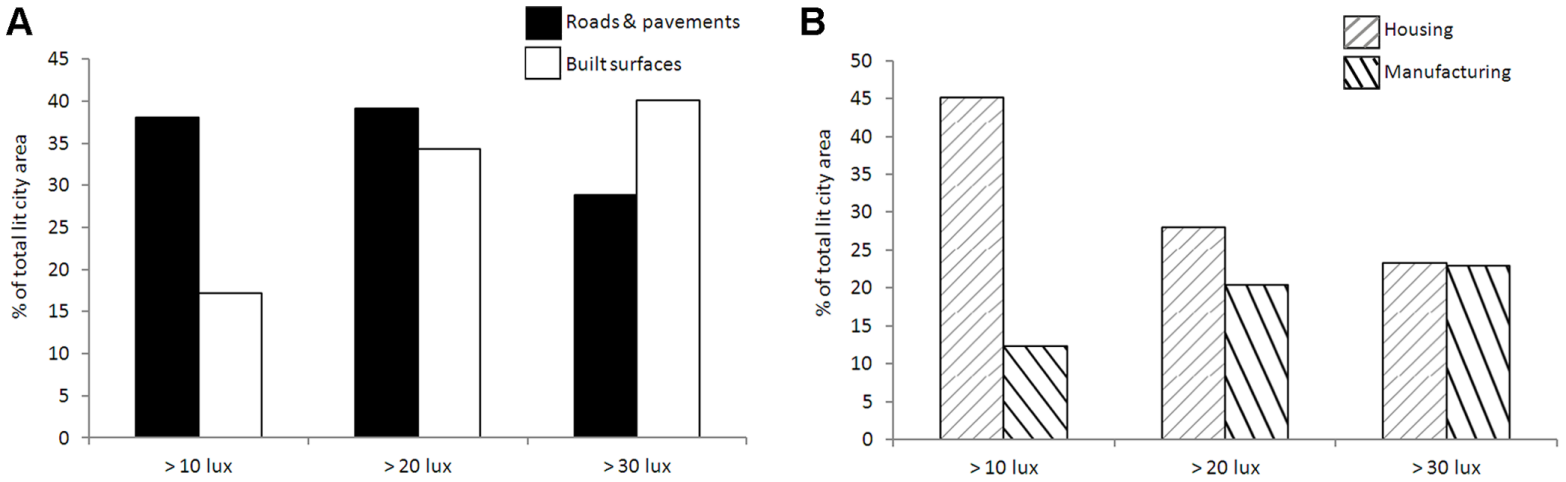
Percentage contribution of land-uses to the total area of the city ≥10, ≥20 and ≥30lx. (A) Roads/pavements and built surface Ordnance Survey MasterMap (OSMM) land-use parcels. (B) Housing and manufacturing National Land Use Database (NLUD) zones.

**Figure 5:**
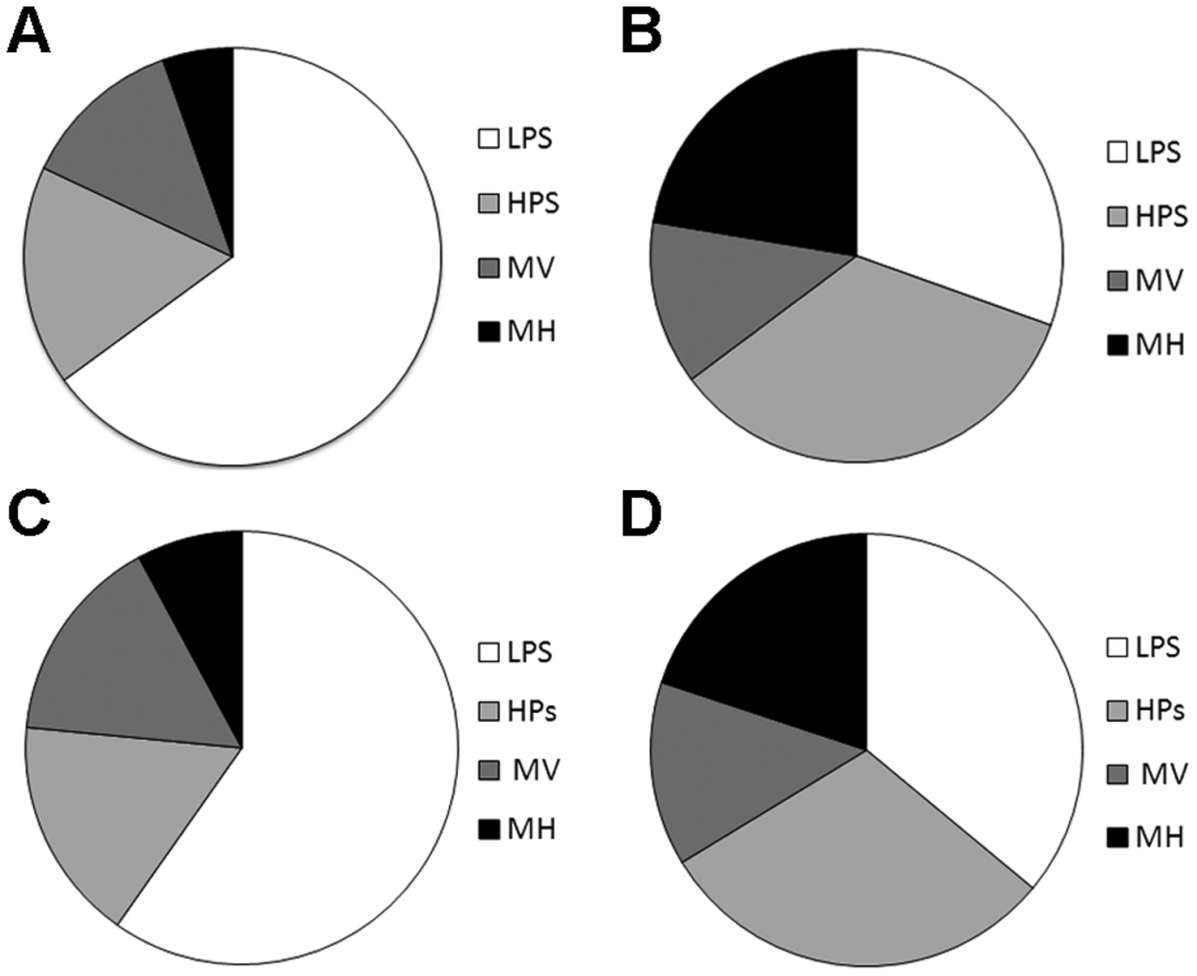
The relative proportions of lamp classes associated with different land-uses. (A) Roads/pavements and (B) other built surface OSMM land-use parcels, located within (C) housing and (D) manufacturing NLUD zones.

The contribution of different OSMM land-use parcels to the total lit surface area within the city varied, with roads/pavements (38%) and other built surfaces such as car parks (17%) contributing the majority of the total area ≥10lx (Fig. 4A). These land-uses are also the main sources of the city’s brighter lighting, although roads/pavements are responsible for a lower percentage (29%) of areas ≥30lx than built surfaces (40%) (Fig. 4A). For NLUD land-use zones, housing (45%) and manufacturing (12%) areas were responsible for the majority of city lighting ≥10lx and approximately equal proportions of areas ≥30lx (Fig. 4B). The distribution of lamps between land-uses is similar to that for lit areas, with the majority of city lamps being associated with OSMM roads/pavements (52%) and other built surface parcels (14%). LPS lamps dominate the lighting of roads/pavements (Fig. 5A), whilst the lamp types associated with other built surface parcels are more evenly spread between LPS, HPS and MH (Fig. 5B). When considering NLUD land-use zones, 55% of city lamps are associated with housing and 11% with manufacturing. Whilst LPS lamps dominate lighting provision within housing zones (Fig. 5C), the lamps in manufacturing zones are more evenly divided between LPS, HPS and MH (Fig. 5D). A more detailed breakdown of lighting and land-use at the city scale is presented for comparison in Table S1.

**Figure 6:**
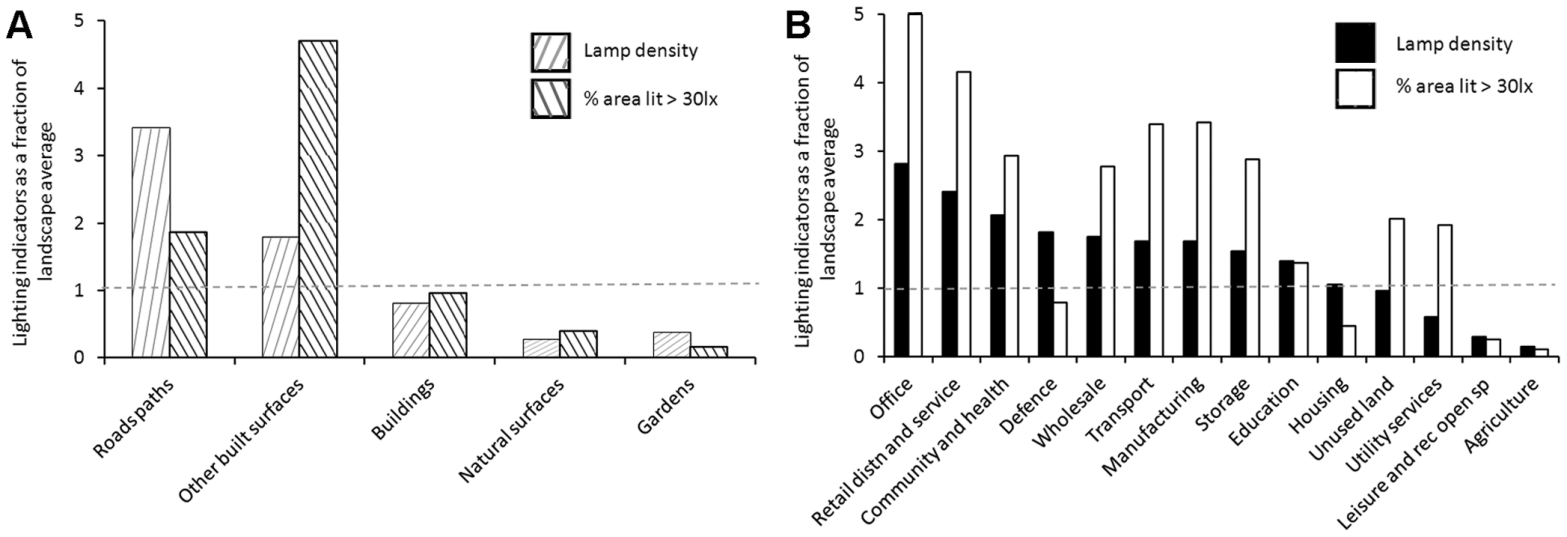
Lamp density and percentage illuminated area (≥30lx) for total city area covered by different land-uses. (A) OSMM land-use parcels and (B) NLUD land-use zones. Values have been standardised, with values >1 indicating abundance is greater than the landscape average.

Although OSMM roads/pavements and other built surface parcels within NLUD housing and manufacturing zones are responsible for the majority of lighting within the city, other land-uses are still intensely lit and therefore may contribute significantly to lighting at local scales (Fig. 6). For example, although office land-use is limited in terms of urban areal extent (<1% of total city area) (Table S1), a 0.01 km^2^ office zone typically has over twice the lamp density and five times the brightly illuminated surface area than the average land-use zone within the city (Fig. 6B). In contrast, a typical 0.01 km^2^ area of housing (which is the dominant land-use zone in the city) has just half the brightly lit area than the city average.

## Discussion

The earth is experiencing a step-change in artificial lighting provision [22], [51], [52]. The replacement and expansion of lighting infrastructure raises the possibility of unintended and broad scale impacts on human health and wellbeing [27], [53] and on ecosystem processes [32]. Although beneficial for many social applications; strong, broad spectrum and extensive lighting at night can interrupt key physiological and behavioural processes for species of plants and animals, including humans [25], [54]. Point sources can be a cause of nuisance due to glare and lighting trespass [45] whilst diffuse sky glow can obscure views of the night sky [27] and eliminate natural cycles in lunar illumination [55]. It is therefore vital that baseline lighting data are collected, against which to measure these changes and to support research into understanding the implications for social and ecological systems. A major advance has been the collection of global data on the extent and magnitude of night lighting [27]. However, many key urban research questions require higher resolution data [36]. Advances in high-specification digital camera technology have now made broad-scale aerial night photography a possibility [40]. For the first time we are able to explore patterns between lighting and urban land-use, using metrics and scales that are relevant to those involved in research, planning and management of cities.

### Built Density

The results of this case study indicate that high built densities are associated with more extensive, brighter and broader spectrum lighting. This has implications for debates about the sustainability performance of the compact city [56]; with the economies that arise from dense urban development [57] potentially being accompanied by greater light pollution. The co-variability between lighting and built density also has implications for studies employing urban-rural gradients [6]; which should take steps to avoid potential confounding effects of lighting on the social or environmental variables of interest. At fine spatial scales (<0.25 km^2^), built density is a poorer predictor of urban lighting. Spatial patterning is therefore nested, with small dark spaces existing even within densely built, brightly lit neighbourhoods. Lighting at fine scales is socially and ecologically relevant and appears to be related to land-use.

### Land-use

The results of our analysis of OSMM parcels and NLUD zones illustrate which land-uses are predominantly responsible for lighting at the city scale and which have a strong local effect. As might be expected, roads/pavements and other built surface parcels within housing and manufacturing zones are responsible for a large proportion of the lamps and brightly lit surfaces within the city, reflecting the role that lighting plays in transport, safety and building security. This suggests that these land-uses should be the target of proactive strategies to reduce light pollution, such as dimming [29], shielding [44] and switch-off [58]. The large-scale replacement of LPS suburban street lighting underway in the UK presents an opportunity to reduce some aspects of light pollution, although it may cause others to increase. The replacement lamps are generally well shielded [28], and their timing and brightness is more easily altered. However, public opposition to switch-off has been considerable [29]. The use of broader spectrum lamps is being driven by the desire for improved colour perception, but may result in greater disturbance to natural processes [25]. Whilst efforts to address current light pollution should continue to focus on suburban street lighting, our research suggests that the security lighting of manufacturing areas may warrant similar attention. These areas occupy a small fraction of the city with relatively few lamps, yet are responsible for a large proportion of bright urban lighting. Concerns have already been raised about light pollution arising from the security lighting of commercial areas [28], [29], [42], and our study provides strong evidence that this is an issue at the city scale. Retail and distribution land-use zones alone account for 11% of all brightly lit surfaces, rising to 34% when manufacturing areas are included; yet these account for just 10% of the city landscape (Table S1). Similar results have been found for central Berlin [40]. In contrast to street lighting, modifications to the positioning and triggering of security lamps may well be more publicly acceptable as well as more effective from a security perspective than current practice [28], [45]. Although they are relatively infrequent land-uses within the case study area; built surfaces within office, retail, transport, community/health, manufacturing and storage zones have lamp and lighting densities that are considerably higher than the landscape average. This has implications for land-use planning as such development may have strong local effects; and future growth in the service and retail sectors has the potential to deliver greater pollution at the city scale.

Whilst useful for raising awareness of the likely lighting implications of development proposals, it is still not known how well these findings transfer between cities and to what extent the lighting characteristics of land-uses described here are fixed. For example, large-scale replacement of lighting infrastructure in the future is likely to result in brighter and broader spectrum lighting [29], [52], although the reverse may be true in some cases [42].

### Future Applications of Urban Lighting Indicators

Although not the focus of this paper, there are a range of additional research and planning applications for the lighting datasets described here. Light maps have the potential to address several topical issues in urban studies and the diversity of applications for remotely sensed lighting data is illustrated by research resulting from the interdisciplinary EU MANTLE project [41]. Similar questions might be addressed using higher resolution data, but as urban relationships and management priorities can be scale dependent, additional questions might also be explored. Higher resolution data have the potential for characterising urban forms [59] and for generating lighting inventories for infrastructure management. They might also be used to scale the results of field studies and research experiments to explore their implications for an entire city. Remotely sensed lighting maps are considered to be unique in their ability to reflect human activity [41]. As research into urban areas tends to underplay their social dimensions [49], the collection and use of lighting maps may help to better integrate these aspects into the modelling of urban systems. From an applied perspective, high resolution mapping would also enable the development of more sophisticated urban lighting masterplans, tailoring lighting to meet the needs of the community at a fine spatial scale and to improve urban lighting governance [45], [60]. Changes to the nature and operation of lighting infrastructure also have the potential to permit considerable financial and carbon savings [34], although some public opposition might be expected [29]. How environmental information is presented can be key to facilitating behavioural change [51] and striking images of cities, neighbourhoods and streets at night could play a useful role in encouraging a broader social debate about lighting, energy and climate change. Combined with analyses such as those presented here, these images may also be useful in challenging false assumptions on the causes and magnitude of artificial lighting and its associated impacts [24].

Artificial lighting can play an important role in shaping urban sustainability, yet little is know about how it varies with land-cover and land-use. In this paper we have demonstrated that aerial night photography can be effective in clarifying these relationships and in challenging conventional approaches to tackling unnecessary or problematic urban lighting.

## Supporting Information

Figure S1 **Ground incident lux plotted against corresponding greyscale pixel value for survey locations within Birmingham.** The equation for the best fit line (y  = 0.0128X^2^+0.2246X +0.8517) was used to reclassify the greyscale raster. R^2^ = 0.9146. A 95% confidence interval is also indicated.
(TIF)

Figure S2 The distribution of greyscale pixel values for known “dark” locations (lit to <1lx).
(TIF)

Figure S3 **CHAID classification tree for lamp classes.** 1 =  low pressure sodium (LPS), 2 =  high pressure sodium (HPS), 3 =  metal halide (MH) and 4 =  mercury vapour (MV). The first discriminating variable was the green to red ratio (G:R 0–1 m) for pixels up to 1 m from the lamp centre. LPS and HPS were then differentiated based on the maximum greyscale pixel value between 2 and 4 m (GS 2–4 m) from the lamp centre. MH and MV were differentiated based on the average blue pixel value up to 1 m from the lamp centre (BL 1 m).
(TIF)

Figure S4 **The results for sampling of lighting metrics at the 0.01** **km^2^ scale.** (A) Percentage area ≥10lx and (B) density of lamps, both plotted against percentage built land-cover.
(TIF)

Figure S5 **Changes in the density of lamp classes along the 1** **km^2^ urban gradient.** (A) MH and LPS lamps and (B) MV and HPS lamps.
(TIF)

Table S1 **Land-uses and lighting metrics for the city of Birmingham.** Land-uses are given as a fraction of total city area, along with their contribution to the total city area lit **≥**30lx and to the total number of city lamps. Two alternative measures of land-use are given; land-use parcels based upon the Ordnance Survey MasterMap (OSMM) (2008) and land-use zones based on the National Land Use Database (NLUD) categories (1995).
(DOC)
