# Supplementary figures and images for: Mapping Lightscapes: Spatial Patterning of Artificial Lighting in an Urban Landscape
Source: PLoS One. 2013 May 6;8(5):e61460. doi: 10.1371/journal.pone.0061460 (PMC3646000; doi:10.1371/journal.pone.0061460)

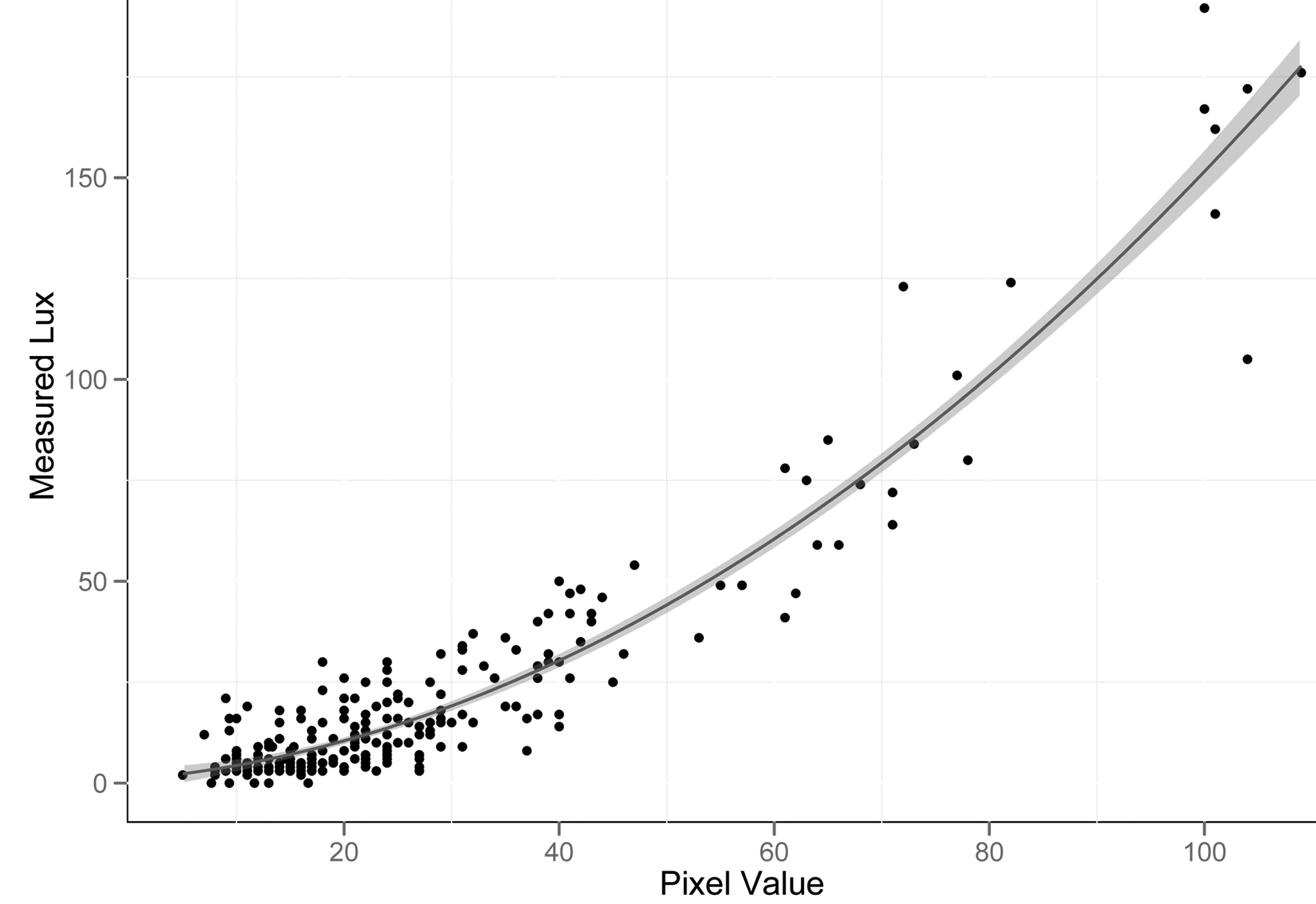

Supplement: Figure S1 — Ground incident lux plotted against corresponding greyscale pixel value for survey locations within Birmingham. The equation for the best fit line (y = 0.0128X2+0.2246X +0.8517) was used to reclassify the greyscale raster. R2 = 0.9146. A 95% confidence interval is also indicated. (TIF) [file pone.0061460.s001.tif]

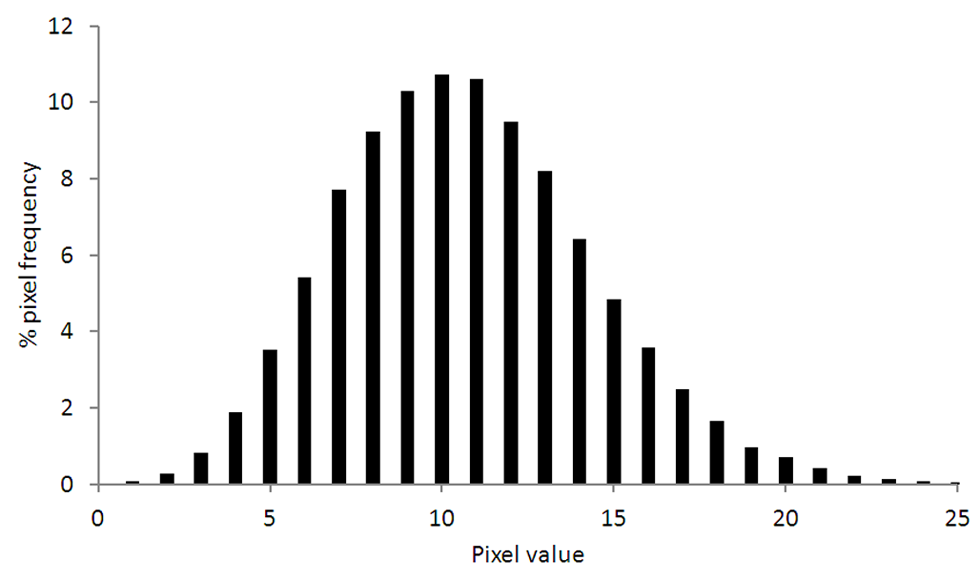

Supplement: Figure S2 — The distribution of greyscale pixel values for known “dark” locations (lit to <1lx). (TIF) [file pone.0061460.s002.tif]

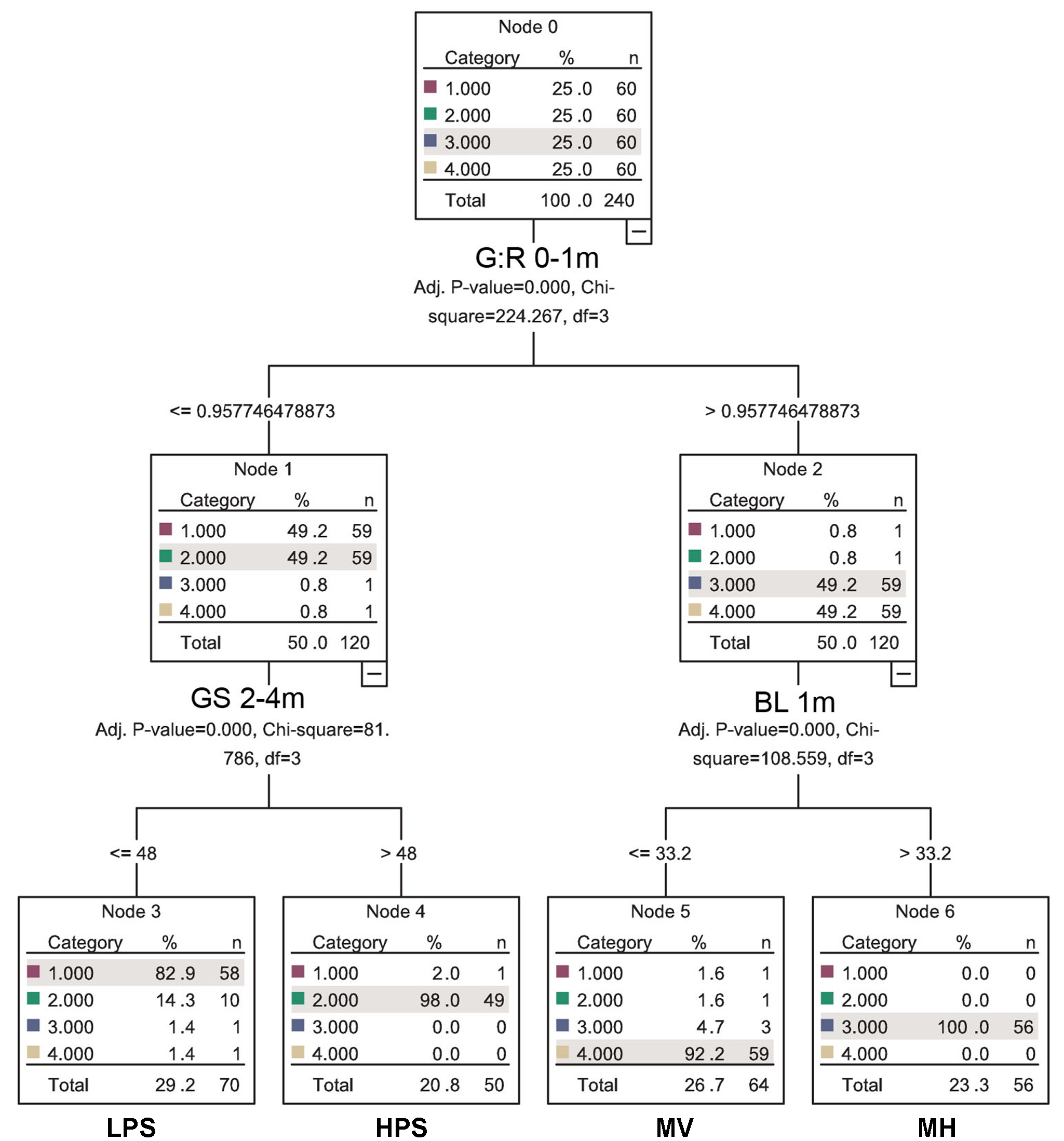

Supplement: Figure S3 — CHAID classification tree for lamp classes. 1 = low pressure sodium (LPS), 2 = high pressure sodium (HPS), 3 = metal halide (MH) and 4 = mercury vapour (MV). The first discriminating variable was the green to red ratio (G:R 0–1 m) for pixels up to 1 m from the lamp centre. LPS and HPS were then differentiated based on the maximum greyscale pixel value between 2 and 4 m (GS 2–4 m) from the lamp centre. MH and MV were differentiated based on the average blue pixel value up to 1 m from the lamp centre (BL 1 m). (TIF) [file pone.0061460.s003.tif]

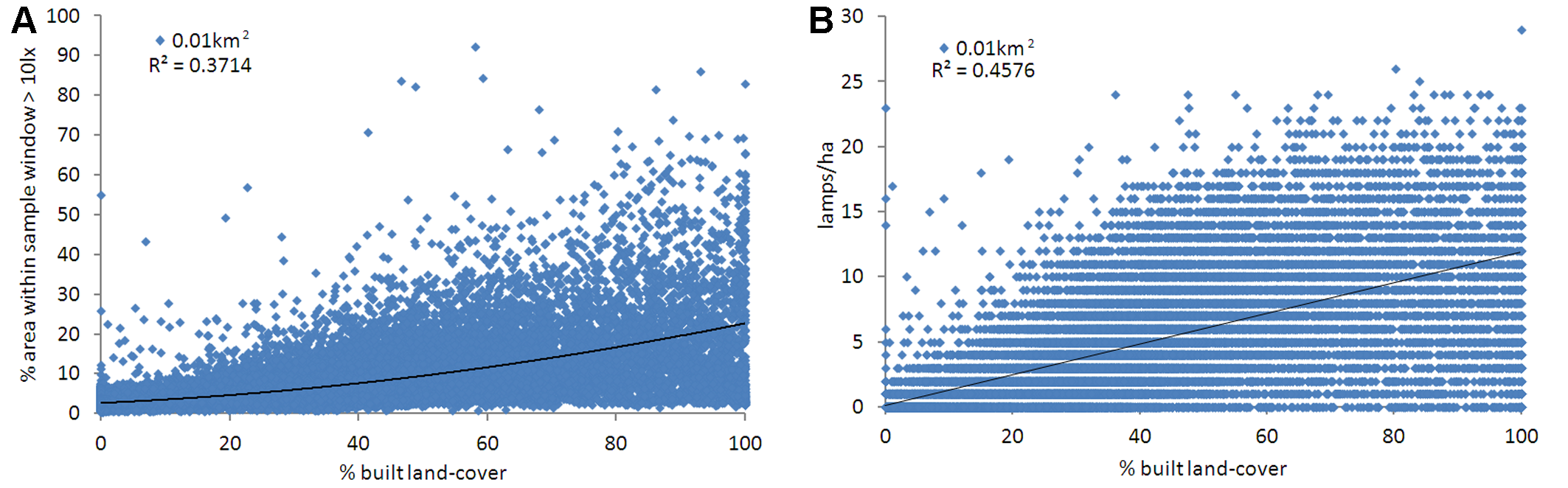

Supplement: Figure S4 — The results for sampling of lighting metrics at the 0.01 km2 scale. (A) Percentage area ≥10lx and (B) density of lamps, both plotted against percentage built land-cover. (TIF) [file pone.0061460.s004.tif]

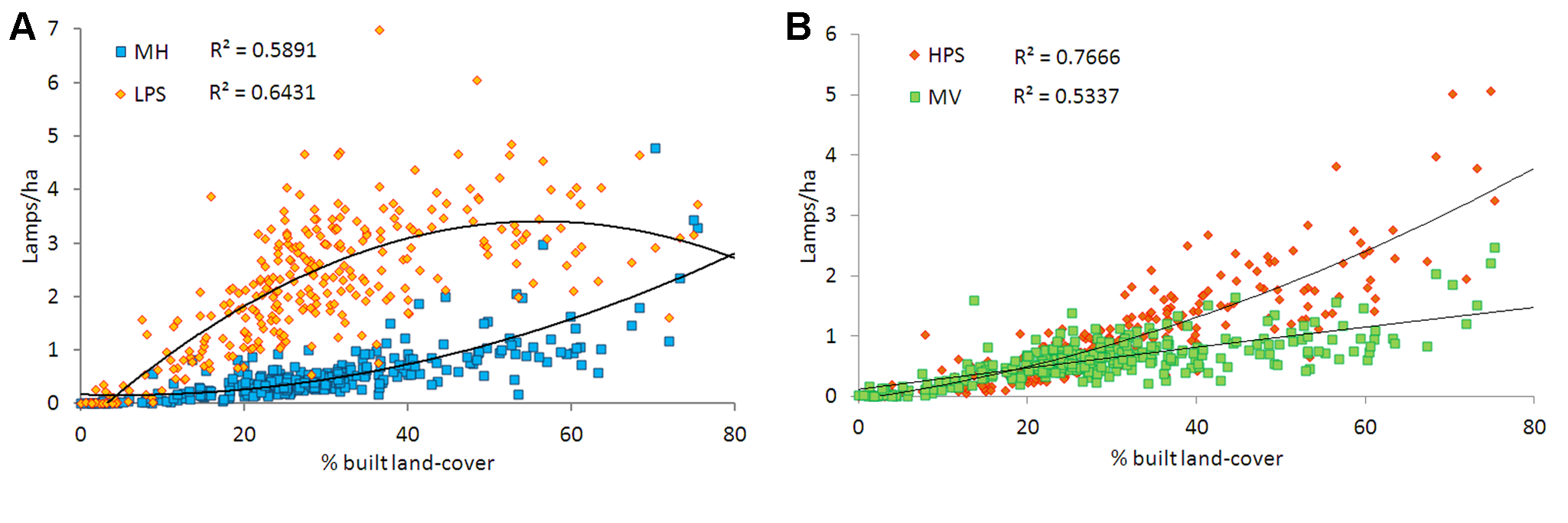

Supplement: Figure S5 — Changes in the density of lamp classes along the 1 km2 urban gradient. (A) MH and LPS lamps and (B) MV and HPS lamps. (TIF) [file pone.0061460.s005.tif]
