# Supplementary material for: Mapping Lightscapes: Spatial Patterning of Artificial Lighting in an Urban Landscape
Source: PLoS One. 2013 May 6;8(5):e61460. doi: 10.1371/journal.pone.0061460 (PMC3646000; doi:10.1371/journal.pone.0061460)
Supplement: Table S1 — Land-uses and lighting metrics for the city of Birmingham. Land-uses are given as a fraction of total city area, along with their contribution to the total city area lit ≥30lx and to the total number of city lamps. Two alternative measures of land-use are given; land-use parcels based upon the Ordnance Survey MasterMap (OSMM) (2008) and land-use zones based on the National Land Use Database (NLUD) categories (1995). (DOC) [file pone.0061460.s006.doc]

| **Classification type** | **Land-use class** | **Land-use as % of total city area** | **% contribution to total city lighting ≥ 30lx** | **% contribution to total city lamps** |
| --- | --- | --- | --- | --- |
| OSMM | Natural land-covers | 33 | 13 | 9 |
|  | Gardens | 29 | 5 | 11 |
|  | Roads & Pavements | 15 | 29 | 53 |
|  | Buildings | 14 | 13 | 11 |
|  | Other built surfaces | 9 | 40 | 15 |
| NLUD | Housing | 52 | 23 | 55 |
|  | Leisure/recreational open space | 16 | 4 | 4 |
|  | Manufacturing | 7 | 23 | 11 |
|  | Agriculture | 7 | 1 | 1 |
|  | Transport | 4 | 12 | 6 |
|  | Education | 4 | 5 | 5 |
|  | Utility services | 3 | 5 | 1 |
|  | Retail distribution and services | 3 | 11 | 6 |
|  | Unused land | 2 | 4 | 2 |
|  | Community and health | 2 | 6 | 4 |
|  | Wholesale | 1 | 2 | 1 |
|  | Office | 0.5 | 3 | 1 |
|  | Storage | 0.2 | 1 | 0.3 |
|  | Defence | 0.1 | 0.1 | 0.2 |
